# Supplementary material for: High-Risk Early-Stage Endometrial Cancer: Role of Adjuvant Therapy and Prognostic Factors Affecting Survival
Source: Cancers (Basel). 2025 Jun 19;17(12):2056. doi: 10.3390/cancers17122056 (PMC12190486; doi:10.3390/cancers17122056)

Table S1. Treatment outcome by adjuvant treatment.

	Total		Death		Peritoneal recurrence		Any recurrence		Loco-regional recurrence		Distant metastasis		3yrOS	3yrDFS	3yrLRRFS	3yrDMFS
	No	%	No	%	No	%	No	%	No	%	No	%	%	%	%	%
All patients	106	100	21	19.8	18	17.0	37	34.9	22	20.8	30	28.3	86.0	65.8	79.6	72.1
Adjuvant treatment																
Any treatment	70	66.0	8	7.5	10	9.4	21	19.8	10	9.4	17	16.0	90.8	70.5	88.3	76.3
Chemotherapy alone	14	13.2	3	2.8	2	1.9	3	2.8	1	0.9	3	2.8	84.4	77.1	92.3	76.6
Radiotherapy alone	37	34.9	2	1.9	5	4.7	11	10.4	6	5.7	7	6.6	97.3	69.3	86.2	80.5
CCRT	2	1.9	0	0	0	0	0	0	0	0	0	0.0	100.0	100.0	100.0	100.0
Sequential CTx-RTx	13	12.3	1	0.9	2	1.9	4	3.8	1	0.9	4	3.8	92.3	68.4	92.3	68.4
Sequential RTx-CTx	2	1.9	1	0.9	1	0.9	2	0.9	1	0.9	2	1.9	50.0	50.0	100.0	50.0
Sandwich treatment (CTx-RT-CTx)	2	1.9	1	0.9	0	0	1	0.9	1	0.9	1	0.9	50.0	50.0	50.0	50.0
Observation	36	34.0	13	12.3	8	7.5	16	15.1	12	11.3	13	12.3	78.7	58.0	63.9	66.1

OS, overall survival; DFS, disease free survival; LRRFS, locoregional recurrence free survival; DMFS, distant metastasis free survival; CCRT, concurrent chemoradiation; CTx, chemotherapy; RTx, radiotherapy

Table S2. Distant metastasis site by histology.

Distant metastasis site (N)			
	Total	NEEC	G3 EEC
Peritoneal seeding	14	9	5
Lung	13	9	4
Abdominal lymph nodes	7	4	3
Mediastinal lymph nodes	5	5	0
Liver	5	3	2
Brain	2	1	1
Bone	1	1	0
Spleen	1	0	1

NEEC, non-endometrioid endometrial cancer; G3 EEC, grade 3 endometrioid carcinoma

Table S3. Treatment outcome by histology.

	Any Progression		Locoregional failure		Distant metastasis	
	N	%	N	%	N	%
NEEC (n=60)	21	35.0%	11	18.3%	19	31.7%
SC (n=23)	11	47.8%	6	26.1%	10	43.5%
CC (n=9)	4	44.4%	2	22.2%	3	33.3%
CS (n=16)	5	31.3%	2	12.5%	5	31.3%
Undifferentiated (n=3)	1	33.3%	1	33.3%	1	33.3%
Mixed (n=9)	0	0.0%	0	0.0%	0	0.0%
Endometroid G3 (n=46)	16	34.8%	11	23.9%	11	23.9%

NEEC, non-endometrioid endometrial cancer; SC, serous carcinoma; CC, clear cell carcinoma; CS, carcinosarcoma

Figure S1. Kaplan-Meier curve of (A) Overall Survival, (B) Disease-free survival, (C) Locoregional recurrence free survival, and (D) Distant metastasis free survival by risk groups.

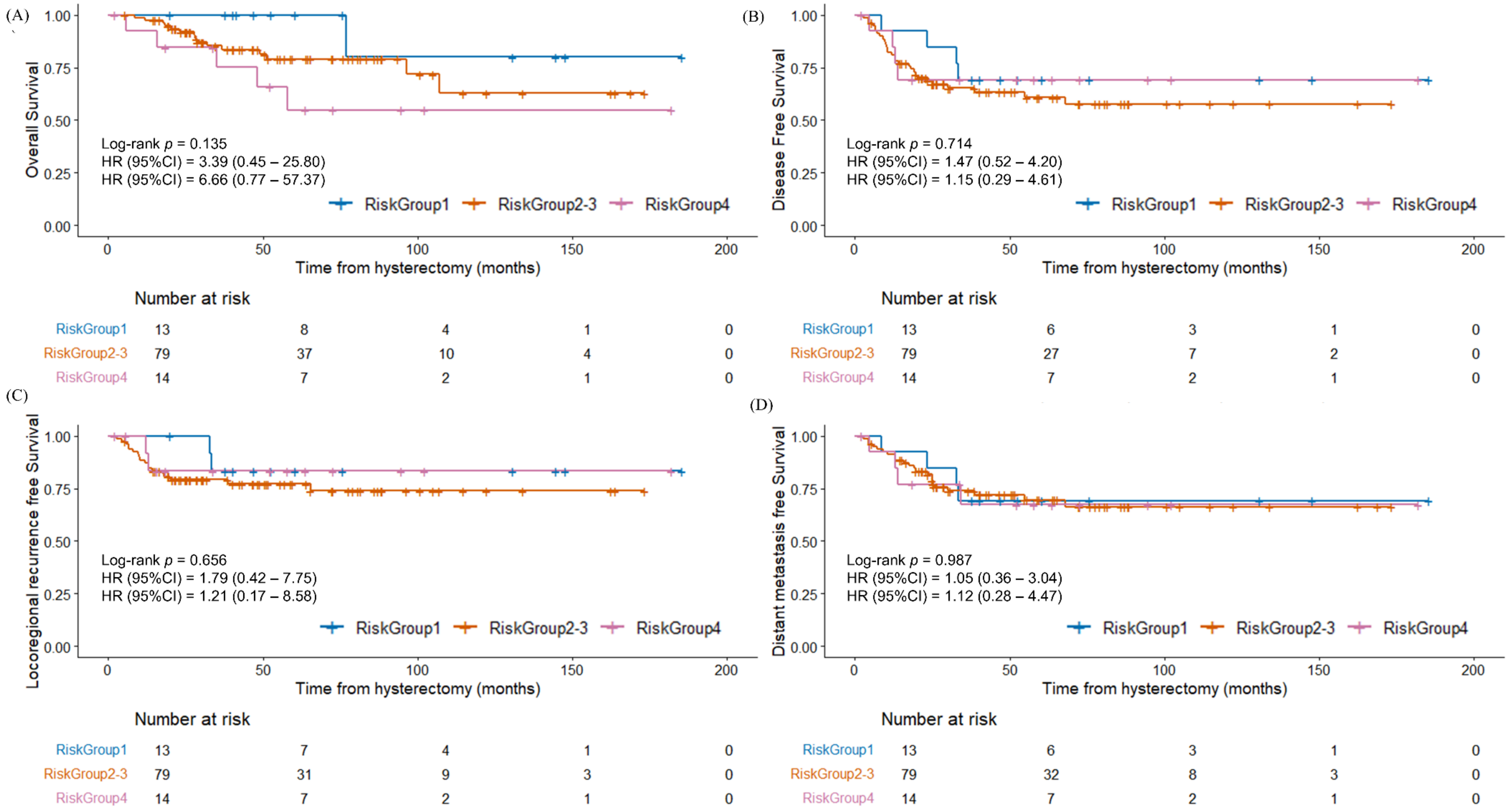

Figure S2. Kaplan-Meier curve of (A) Overall Survival, (B) Disease-free survival, (C) Locoregional recurrence free survival, and (D) Distant metastasis free survival by adjuvant therapy stratified by risk groups (risk group 1, risk group 2-3, and risk group 4).

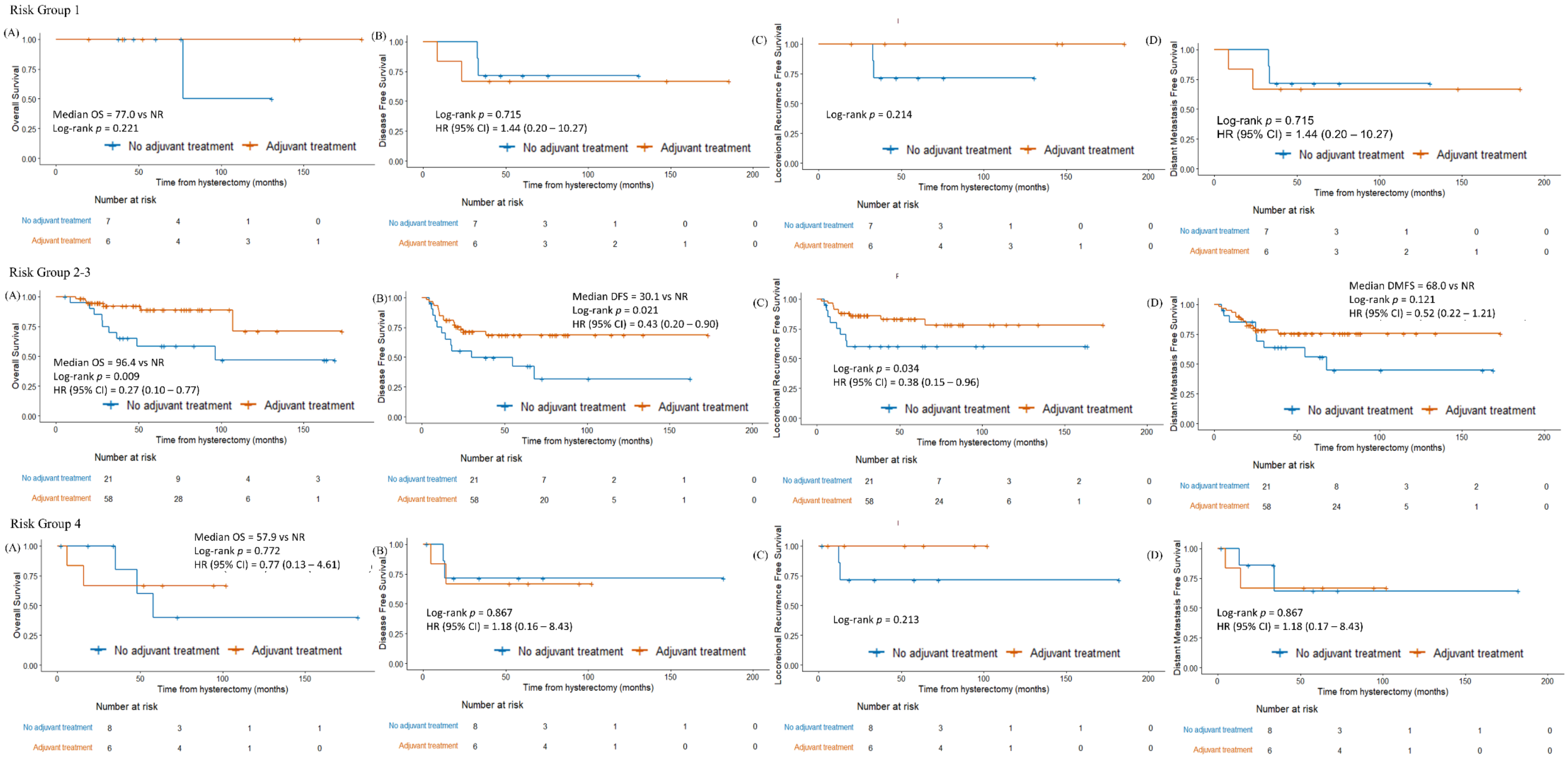

Supplement: Supplementary file 1 [file cancers-17-02056-s001.zip › cancers-3641476-supplementary.pdf]
